# Bromine in the Treatment of Diarrhœa and Dysentery

**Published:** 1864-12

**Authors:** H. F. Gilbert

**Affiliations:** Executive Officer


### CHICAGO MEDICAL EXAMINER.

N. S. DAVIS, M.D., Editor.

VOL. V. DECEMBER, 1864. NO. 12.

### Original Contributions.

ARTICLE XLI.

### BROMINE IN THE TREATMENT OF DIARRHŒA AND DYSENTERY.

Office Prison Hospital,

*Rock Island, Ill. November* 14, 1864.

Doctor:—I have the honor to report the result of a course of experimenting, the object of which was the discovery of a remedy for the cure of chronic diarrhœa and dysentery, as they occur in camp-life.

September 5, 1864, I organized a ward capable of accommodating 43 patients, and up to the 20th of October, 90 have been admitted; 47 were cases of chronic diarrhœa, 31 acute dysentery, and 12 acute diarrhœa.

Treatment.

℞. Bromine, ---------------------------------- qtts. vj.
Bromide of Potassium, ---------------- grs. xv.
Aqua, ------------------------------------ ℥j. M.

I administered one drachm every two hours during the day, without regard to stage, complicating character of discharge, or constitutional symptoms, with the following results:—Returned to barracks, cured, 79; remaining in ward, 10; died, 1. The remaining 10 are suffering from chronic diarrhœa, complicated with scurvy; very much emaciated, and will not be able for duty within sixty days; and all such cases are ordered South for exchange. I am of the opinion that they would finally recover, were they kept on the bromine treatment. The *post mortem* made on the body of the case resulting in death, shows its complications; and the difficulty of treating such cases is apparent to the most casual observer.

William Baily, Private, Co. B, 2d Ky. Cav. transferred into ward, October 11, 1864; died October 19, 1864.

Sectio cadaveris; 36 hours after death. Body:—Greatly emaciated. Thorax:—Both lungs were numerously scattered over the whole surface with miliary tubercles, but somewhat aggregated at the apices, both pleuræ adherent throughout by strong adhesions; the parenchyma between the turbercles was much indurated by pneumonia, of a grey color, from pigmentary deposit; heart very large, abnormal, and fatty.

Abdomen:—The liver enlarged, and greatly altered in shape, of a dusky color, and on section presenting semi-translucent edges; stomach ulcerated to some extent; mucous membrane of large intestines was extensively ulcerated; the bowels thickened and disorganized; small intestines were œdematous, and the coats generally thickened; about the middle-third of the jejunum was found an intussusception of about two inches in length; immediately above and below were two worms (lumbricoid) of different size; the smaller above, and the larger below; beneath the invagination, the gut was greatly congested; other organs healthy.

I found the acute cases, both of diarrhœa and dysentery, recover with greater rapidity than did the chronic; the progress of the latter, in my opinion, was retarded in a great measure by the too frequent use or administration of the remedy, for in the extreme emaciated cases, the medicine would cause a slight nausea of the stomach, thereby preventing the patient from taking the proper amount of nourishment necessary to sustain life. But when administered at long intervals, no such symptoms occurred, as shown from experiments outside of my ward. The digestion improved, griping and tenesmus subsided, and the discharges gradually became more natural, until the patient was entirely restored to health. It is proper to state that the chronic diarrhœa cases were the worst that could be selected from the hospital; most of them were of over 12 months standing, greatly emaciated, tongue dry, and contents of the bowels passing off, as they lay in bed, without an intermission.

In those cases, complicated with chronic inflammation of the lungs and lining membrane of bronchial tubes, and even where the lung was dense, solid, and impervious to air, in some part (which is more or less the condition of these extreme emaciated diarrhœa subjects) I found the pains of the chest, dyspnœa, together with the troublesome cough entirely disappear in a very short time, and the patient expressing himself as "feeling like a new man."

Whether the bromine acts directly upon the diseased pulmonary tissue by inhalation, or in some other way, remains to be ascertained. As it is exceedingly vaporable, I am inclined to the opinion, that its good results are due to the direct application of the vapor to the tissue involved; a theory in the treatment of chest diseases, I confess I am surprised at the profession viewing with so much indifference. Be this right or wrong, certain it is, I have been astonished at the happy results in such cases as the above, treated with bromine.

Bromine is a new remedy, its status as yet remains unknown or unsettled, and we have but imperfect data from which to draw our conclusions; still I am impelled from personal observations and experience to assert to the medical department, that in bromine we have an agent, certain in its effects to cure all cases of diarrhœa and dysentery, I care not of how long standing, if the patient has sufficient vitality left to sustain life five or six days; a perfect restoration to health may be relied upon, unless the disease is complicated with some serious malady.

I am aware that I am making a sweeping assertion, yet I doubt not that the time will come when the medical profession, both in America and Europe, will verify what I am now saying. I herewith append a note from Dr. SALTER, who has been using bromine in the barracks.

*Rock Island Barrcks, Illinois, Oct.* 12, 1864.

Dr. H. F. Gilbert,

Dear Sir:—Since I had the pleasure of listening to your remarks on the use of bromine in dysentery and diarrhœa, I have been testing for myself the efficacy of that medicine, in the two diseases above mentioned. I have been prescribing it in the prison barracks, where the situation and attending circumstances are by no means favorable for the administration of any remedy, and least of all in diarrhœa and dysentery, notwithstanding, I have found it in many instances of the above complaints to have answered a most excellent purpose, and I believe it to be a very valuable remedy. I do not think, that I ever tried any one article of materia medica, which has proven so satisfactory under all circumstances. There have been several cases apparently not benefitted by its administration, thus proving it not a specific; yet I cannot say that under more favorable circumstances, it might not have been more satisfactory, even in these, and I would here remark that these cases were not relieved by any previous course of treatment, and were subsequently sent to hospital. I would say, in conclusion, that in the treatment of these two diseases, I have found in the bromine a most valuable remedy, in the administration of which, in many severe cases, I have been both surprised and pleased with its efficacy, and deem it well worthy the attention of the profession. Hoping that in your efforts to fathom the full force of bromine, and its applicability to the various diseases now being tested by you, the fullest success may attend.

H. F. SALTER, A.A.S., U.S.A.

In conclusion, I would say that the medical descriptive lists of the above cases, have been carefully kept, and in due time they will be published to the world, that the medical profession may judge of the character of the cases treated, and the power of bromine in effecting a most astonishing cure.

I am sir, very respectfully, your obedient servant,

H. F. GILBERT, *Executive Officer.*

Surg. C. S. Tripler, U.S.A.,

*Medical Director, Northern Department.*

